# Development and validation of a brief diabetic foot ulceration risk checklist among diabetic patients: a multicenter longitudinal study in China

**DOI:** 10.1038/s41598-018-19268-3

**Published:** 2018-01-17

**Authors:** Qiuhong Zhou, Min Peng, Lihuan Zhou, Jiaojiao Bai, Ao Tong, Min Liu, In I Ng, Yuxia Cheng, Yunmin Cai, Yujin Yang, Yilian Chen, Suwen Gao, Zhong Li, Xiaoai Fu, Minxue Shen, Jianglin Zhang, Xiang Chen

**Affiliations:** 1 Department of Outpatient, Xiangya Hospital, Central South University, Changsha, Hunan China; 2 Xiangya School of Nursing, Central South University, Changsha, Hunan China; 3 Department of Nursing, Huadong Hospital Affiliated to Fudan University, Shanghai, China; 4 Department of Endocrinology, Air Force General Hospital of PLA, Beijing, China; 5 Department of Endocrinology, West China Hospital of Sichuan University, Chengdu, Sichuan China; 6 Diabetes Care Center, Kiang Wu Hospital, Macau, China; 7 Department of Endocrinology, The 306th Hospital of PLA, Beijing, China; 8 Wound Healing Center, Jinshan Hospital Affiliated to Fudan University, Shanghai, China; 9 Department of Nursing, The Second Affiliated Hospital of Nanchang University, Nanchang, Jiangxi China; 10 Department of Gastroenterology, Puai Hospital, Huazhong University of Science and Technology, Wuhan, Hubei China; 11 Department of Endocrinology, Handan Central Hospital, Handan, Hebei China; 12 Department of Nursing, Qinghai Red Cross Hospital, Xining, Qinghai China; 13 Department of Endocrinology, Xiangya Hospital, Central South University, Changsha, Hunan China; 14 Department of Dermatology, Xiangya Hospital, Central South University, Changsha, Hunan China; 15 Hunan Key Laboratory of Skin Cancer and Psoriasis, Changsha, Hunan China

## Abstract

The study aims to develop and assess and validate a brief diabetic foot ulceration risk checklist among diabetic patients through a longitudinal study. Patients who had diabetes mellitus and had no foot ulceration and severe systematic disorders were recruited from eleven tertiary hospitals in nine provinces or municipalities of China. Internal consistency reliability, construct validity, concurrent validity, item property, and measurement invariance of the tool were assessed. The predictive capability of the tool was validated by the follow-up data using the receiver operating characteristic curve. At baseline, 477 valid cases were collected. Twelve items were remained after initial selection. Cronbach’s alpha was 0.56. Confirmatory factor analysis showed that the model had acceptable goodness-of-fit yet local dependency between two items. Item response theory showed that most items had acceptable discrimination and difficulty parameters. Differential item functioning showed that tool had measurement invariance. 278 were followed up one year after the baseline. Follow-up showed that one-year incidence of ulceration among the patients was 3.6%, and the area under the receiver operating characteristic curve was 0.77 (95% confidence interval: 0.61–0.93). The cut-off point of the tool was 4, when sensitivity and specificity were 0.62 and 0.75 respectively. The checklist has good psychometric properties according to mixed evidences from classical and modern test theory, and has good predictive capability.

## Introduction

Diabetes and its complications have become significant public health problems. Diabetic foot is a severe chronic diabetic complication that consists of lesions in deep tissues associated with neurological disorders and peripheral vascular diseases in the lower extremities^1^. Diabetic foot ulceration (DFU) is the leading cause (32.6%) of chronic cutaneous wounds in China, followed by trauma and burns (23.8%)^2^. DFU is associated with increased disability rate and mortality, and is the main cause of hospitalization among diabetic patients. In China, the annual incidence of ulceration and amputation was 8.1% and 5.1% among diabetic patients, respectively, and the fatality rate among patients with ulceration was 14.4%^3^. DFU is also associated with heavy medical burden. The annual average cost of DFU is 8,659 US dollars per patient globally^4^. The average cost of hospitalization for DFU is 17,183 *yuan* in China, and the average length of stay is 18 days^5^. Therefore, the International Diabetes Foundation is increasing awareness of diabetic foot problems owing to substantial social and medical burdens^6^.

Gavin’s score, a seven-item short scale with different weights for each item^7^, has been ubiquitously used to evaluate the risk of developing DFU in the clinical practice in China^8,9^. The total score is 13 and patients are categorized into three subgroups: low risk, moderate risk and high risk. However, the tool has not been validated yet, and the property of the tool is unknown; and the weights of items were subjective without scientific evidences. Recently, the International Working Group on the Diabetic Foot (IWGDF) published a guidance for the prevention and management of foot problems in diabetes^10^. A checklist of risk factors for foot ulceration was provided; but quantitative assessment was also not available. For the purpose of better disease management for diabetic patients, especially in multidisciplinary treatment setting, a valid and precise tool to assess the risk of DFU is needed in clinical practice. Therefore, we developed and validated a brief checklist using classical and modern test theory among diabetic patients from different regions of China. The checklist included well-recognized risk factors for DFU. This tool is different from Gavin’s scale in several aspects: (1) process of tool development and validation; (2) dimensionality; (3) rationality of scoring weights; (4) different items and method of measurement; (5) purpose of use (multidisciplinary, quantitative, predictive assessment).

## Materials and Methods

### Study design and participants

The baseline data of a prospective study was used to validate the tool. Eleven tertiary hospitals from nine provinces or municipalities of China (North: Beijing, Hubei; West: Sichuan, Qinghai; East: Shanghai, Jiangxi; South: Macau, Hunan, Hubei) were selected through non-probability sampling. Diabetic patients were recruited from November to December 2015 and followed up one year after baseline. Inclusion criteria were: (1) had diabetes mellitus diagnosis by WHO criteria of 1999; (2) no DFU; (3) speaking Chinese or English (for patients from Macau); (4) agreed to participate with written informed consent. Exclusion criteria were: (1) severe cognitive impairment; (2) complicated with mental disorder such as depression. Patients were recruited by research nurses.

### Tool development

Based on literature review and expert advice, common risk factors for DFU^10^ were selected. Tool development was performed in four phases:

**Phase I:** Programmed decision processing was used to develop the scales by a nominal group. A total of 37 items was drafted by interviewing the nominal group.

**Phase II:** Individual questions were edited and redundant questions were eliminated by a focus group consisting of 10 experts of dermatologist (n = 3), endocrinologist (n = 3), epidemiologist (n = 2) and nurse (n = 2). An initial pool of 20 items was derived (Table 1). The responses to all items were scored on a true-or-false scale. Items were categorized into five dimensions: (1) neuropathy and vasculopathy; (2) structural deformity and associated changes; (3) course of disease and comorbidity; (4) ulcer history and general skin change; and (5) fungal infection of skin and toenail.

**Table 1:** Brief diabetic foot risk checklist.

No.	Items^*^	Removed items
1	Smoking	Yes
2	Course of diabetes ≥10 years	
3	Having nephropathy	
4	Having retinopathy	
5	Previous ulceration or amputation	
6	Presence of skin changes (damage, redness, or edema)	
7	Presence of hyperpigmentation	Yes
8	Dry foot skin	Yes
9	Presence of rhagades	Yes
10	Fungal infection of foot skin	
11	Presence of callus or corn	
12	Structural deformity of foot	
13	Fungal toenail	
14	Ingrown toenails	Yes
15	Onchogryposis	Yes
16	Wearing uncomfortable shoes	Yes
17	Wearing uncomfortable socks^.^	Yes
18	Abnormal foot skin temperature	
19	Dorsalis pedis pulse diminution	
20	Loss of protective sensation	

^*^All items were answered in yes-or-no pattern.

**Phase III:** The initial item pool included 20 items. The items were selected by statistical methods as following: (1) *t*-test. Participants were ranked by the score on the scale, and a high- and a low-score group were derived respectively according to percentiles (*P*75 and *P*25). The score of each item was compared using a Student’s *t*-test. Any item with no statistical difference between the two groups was removed. (2) Correlation coefficient. Any item with a Pearson’s correlation coefficient <0.40 with the scale score was removed. (3) Factor analysis. Any item with a factor loading <0.40 was removed. (4) Item response theory. Any item with discrimination parameter <0.5 was removed.

**Phase IV:** The scales were validated among 477 patients with diabetes. The reliability, construct validity, and differential item functioning (DIF) were assessed. DIF examined the ability of the tool to scale different groups of objects onto a common metric.

### Baseline assessment

The tool contains 20 items (Table 1) and demographic information. Smoking, course of the disease, and history of ulceration were patient-reported. Other items were assessed by our multidisciplinary team comprised of endocrinologists, dermatologists, plastic surgeons, enterostomal therapists, and nurses. Retinopathy and nephropathy were determined by doctor’s diagnosis. Skin changes (including damage, redness, edema, hyperpigmentation, dry skin, rhagades, fungal infection, callus and corn) as well as toenail changes (including fungal toenail, ingrown toenails, and onchogryposis) were assessed by research nurses and consultant dermatologists. Structural deformity including talipes cavus, talipes equinus, talipes calcaneovalgus, talipes calcaneovarus, flat foot, hammer toe, hallux valgus, and Charcot’s foot was assessed by nurses. Foot skin temperature was determined by infrared thermometers, and abnormal temperature was defined as dorsal pedal or interdigitalis temperature ≤26 °C, or temperature difference between feet ≥2 °C. Loss of protective sensation was evaluated using the SemmesWeistein 5.07/10 g monofilament. Each positive item will be scored one point.

### Follow-up

Patients were followed up through mobile phone one year after the baseline investigation. Occurrence of DFU during the past one year was asked. When a self-reported foot ulcer case was captured through the follow-up, the patient was asked to provide the medical record (with clinical description of the wound, diagnosis, and treatment) for us to review. DFU was defined as Wagner–Meggit grade I (superficial ulcer) to V (gangrene of entire foot) foot lesion.

### Statistical analysis

After initial item selection, the reliability, structural validity, item properties, and measurement invariance of the new tool were then assessed. First, Cronbach’s alpha was used to assess the internal consistency reliability. Since repeated measures were not conducted, test-retest reliability was not determined.

Second, confirmatory factor analysis (CFA) was performed according to the prior hypothesis on dimensionality. Goodness-of-fit of CFA model was assessed by adjusted goodness of fit index (AGFI), comparative fit index (CFI), and root mean square error of approximation (RMSEA). Value of AGFI and CFI >0.9 and RMSEA <0.08 indicate acceptable goodness of fit^11^. Factor loadings of the items were reported.

Third, item response theory (IRT) was used to evaluate the item performance and precision of the measurements. IRT is a family of associated mathematical models that relate latent traits (ability) to the probability of responses to items in an assessment^12^. We applied a two-parameter logistic IRT model for dichotomous responses. The model includes a difficulty parameter and discrimination parameter for each item. The difficulty parameter is the point on the ability scale that corresponds to a probability of a correct response of 0.5. Items with a discrimination parameter of 0.5 to 2.0 and a difficulty parameter corresponding to a certain region of the ability scale (−3.0 to 3.0) will provide the most information. IRT parameters were estimated using a marginal maximum-likelihood method. Patients’ abilities were estimated using the Bayesian method.

Fourth, measurement invariance of the tool between male and female patients was estimated by DIF. Gender differences of discrimination and difficulty parameters for all items were examined using the chi-square tests.

Last, the scale was validated by the actual occurrence of DFU. Scale score were compared between patients who had DFU and who had not using Student *t* test. Receiver operating characteristic (ROC) curve was plotted, and area-under-the-curve (AUC) was calculated.

Estimation of IRT parameters and ability were performed using the Bock-Aitkin procedure in IRTPRO 3 (Scientific Software International Inc., Lincolnwood). CFA was performed in AMOS 19.0 (Arbuckle JL and SPSS Inc., Chicago, USA). Other analyses were performed using SAS University Edition (SAS Institute Inc., Cary, North Carolina). The significance level for all statistical tests was 0.05.

### Power estimation

Power of test was calculated according to the method involving binormal ROC curve indices based on Taylor series expansions^13^. The power was 94% under the following parameters: number of true patients *N*_*A*_ = 10, area under the ROC curve *θ* = 0.77, range of confidence interval *Δ* = 0.93–0.61 = 0.32, type I error *α* = 0.05 (double side), ratio of standard deviation *B* = 1, ratio of normal and abnormal objects *R* = 26.8 (this ratio was high owing to the low one-year incidence of DFU in our longitudinal observation).

### Ethics statement

This study was conducted according to the guidelines laid down in the Declaration of Helsinki. All procedures involving patients were approved by the institutional research ethics boards of Xiangya Hospital, Central South University (Changsha, China). Written informed consent was obtained from all patients.

## Results

A total of 477 patients from eleven tertiary hospitals in nine provinces or municipalities of China were investigated to validate the tool. The characteristics of the patients are shown in Table 2. After the initial assessment, eight items were removed from the original item pool: hyperpigmentation, dry skin, rhagades, ingrown toenails, onchogryposis, uncomfortable shoes, uncomfortable socks, smoking. Twelve items were selected from the item pool (Table 1). The tool demonstrated a suboptimal Cronbach’s alpha of 0.56. Test-retest reliability was not determined.

**Table 2:** Characteristics of patients.

	N (%)/mean ± standard deviation
Gender	
Male	249 (52.2)
Female	228 (47.8)
Education	
Primary school	129 (27.0)
Middle school	202 (42.3)
College/University/Graduates	146 (30.7)
Occupation	
Farmer	40 (8.4)
Worker	79 (16.6)
Civil servant	67 (14.0)
Liberal professions	58 (12.2)
Other	233 (48.8)
Type of diabetes	
1	16 (3.3)
2	438 (91.8)
Unspecified	23 (4.8)
Age (years)	61.1 ± 13.7
Smoking (years)	5.8 ± 11.9
Course of disease (years)	9.3 ± 7.5
Fasting blood glucose (mmol/L)	8.5 ± 3.3
Postprandial blood glucose (mmol/L)	12.2 ± 11.1
Glycosylated hemoglobin HBA1c (%)	7.9 ± 2.2

**Figure 1:**
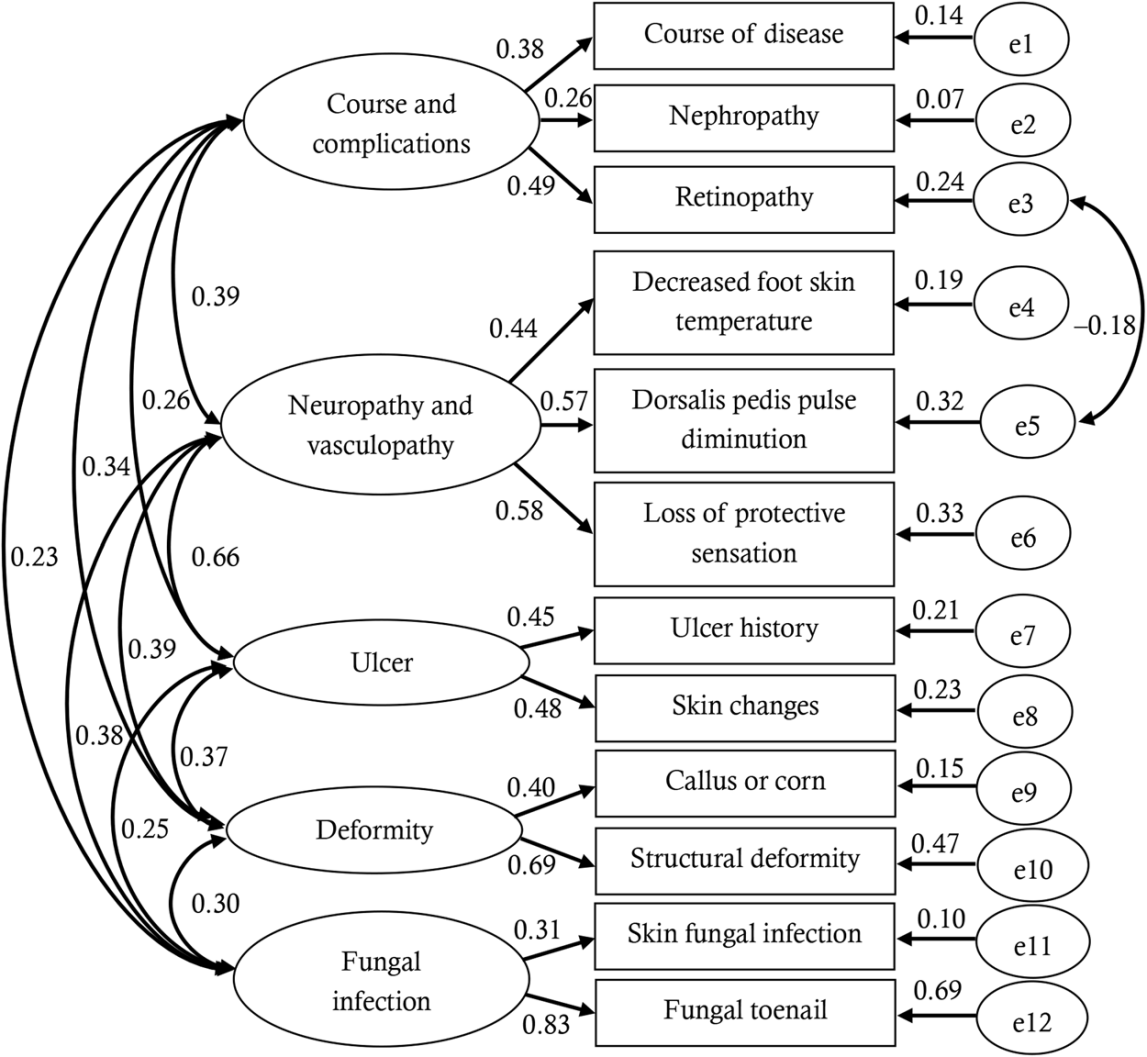
Confirmatory factor analysis model. Local dependency was identified between two items (retinopathy and dorsalis pedis pulse diminution). Except nephropathy and fungal infection of foot skin, most items had acceptable factor loadings on their dimensions.

According to the initial CFA model, local dependency was identified between two items: retinopathy and dorsalis pedis pulse diminution. The structure of CFA model and factor loadings are shown in Fig. 1. Nephropathy and fungal infection of foot skin had suboptimal factor loadings on corresponding dimensions. Overall, the model had acceptable to good goodness-of-fit (AGFI = 0.953, CFI = 0.905, RMSEA = 0.041).

A two-parameter logistic IRT model was used to assess item performance. Discrimination (*a*_*i*_) and difficulty parameters (*b*_*i*_) are shown in Table 3. Except nephropathy and retinopathy, most items had moderate or high discrimination power. Loss of protective sensation had the highest discrimination parameter among all items.

**Table 3:** Item response model parameters and differential item functioning.

	*a*_*i*_ (SE)	*b*_*i*_ (SE)	*P* for DIF^*^	
			*a*_*i*_	*b*_*i*_
Course of disease	0.56 (0.14)	0.66 (0.22)	0.73	0.60
Nephropathy	0.27 (0.19)	7.81 (5.42)	0.43	0.01
Retinopathy	0.43 (0.14)	2.22 (0.73)	0.10	0.14
Previous ulceration	1.45 (0.33)	2.25 (0.34)	0.27	0.28
Presence of skin changes	1.31 (0.28)	2.10 (0.31)	0.92	0.34
Fungal infection of skin	0.54 (0.16)	2.58 (0.72)	0.15	0.43
Callus/corn	0.82 (0.21)	2.56 (0.55)	0.87	0.15
Structural deformity	0.98 (0.20)	1.58 (0.26)	0.78	0.01
Fungal toenail	0.93 (0.17)	0.77 (0.15)	0.03	0.47
Abnormal foot skin temperature	1.27 (0.24)	1.37 (0.19)	0.02	0.87
Dorsalis pedis pulse diminution	1.43 (0.25)	0.68 (0.11)	0.24	0.34
Loss of protective sensation	1.69 (0.27)	0.88 (0.11)	0.96	0.96

*a*_*i*_: discrimination parameter; *b*_*i*_: difficulty parameter; SE: standard error. DIF: differential item functioning.

^*^P values of chi-square tests to examine differential item functioning with respect to gender-specific discrimination (*a*_*i*_) and difficulty (*b*_*i*_) parameters.

**Figure 2:**
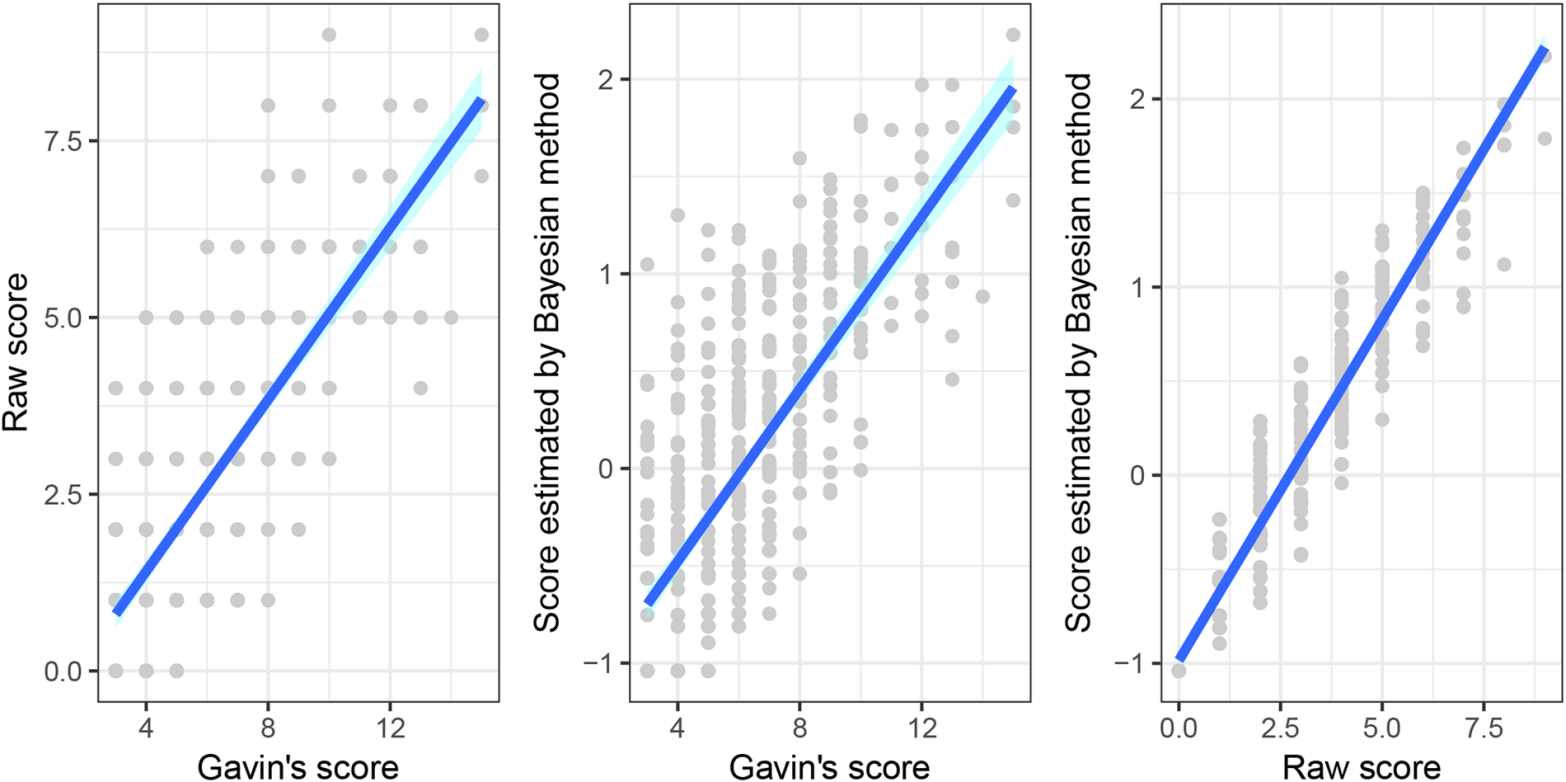
Scatter plots for Gavin’s score, raw score and the Bayesian estimates of ability. Gavin’s score was highly associated with both raw test score (Pearson’s r = 0.76) and Bayesian score (Pearson’s r = 0.73). Bayesian score was the Bayesian estimates of patients’ ability (namely the risk of developing diabetic foot); their posterior standard deviations served as standard errors. Bayesian score obeys Gaussian distribution with mean = 0 and standard deviation = 1.

Concurrent validity was assessed by Gavin’ score (Fig. 2). Gavin’s score was highly associated with the raw score of the test (Pearson’s correlation r = 0.76, *P* < 0.001) as well as Bayesian estimates of patients’ ability (r = 0.73, *P* < 0.001). DIF showed that most items had measurement invariance between male and female patients (Table 3). Only item-level DIF was observed. Overall, the tool could scale male and female patients onto a common metric.

**Figure 3:**
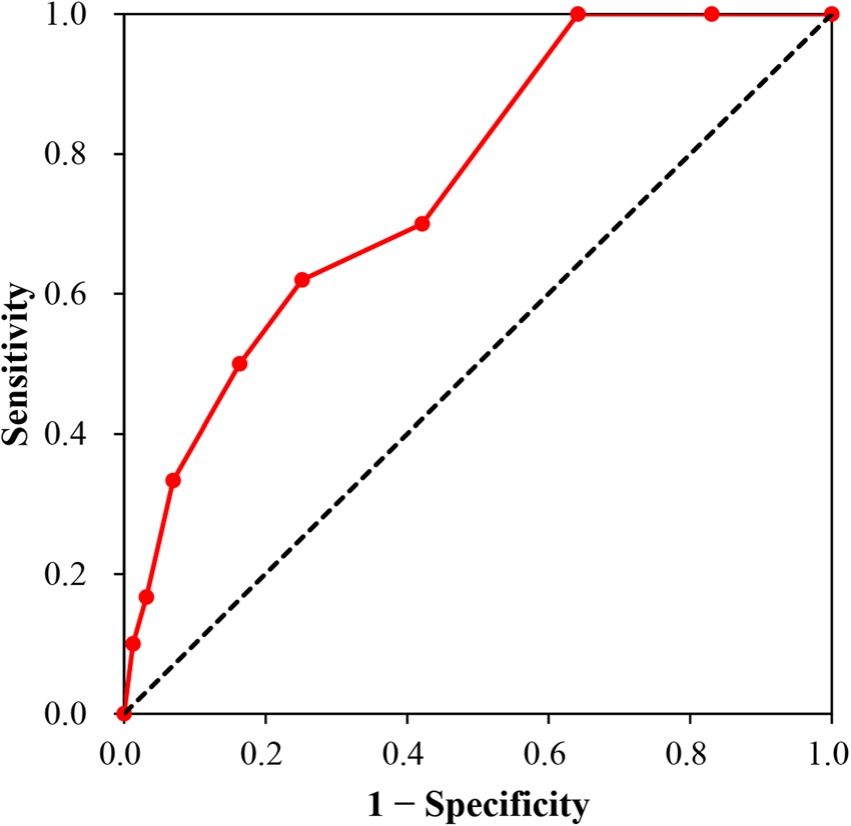
Receiver operating characteristic curve of the scale score to predict diabetes foot ulceration. Area-under-the-curve was 0.77 with 95% confidence interval of 0.61 to 0.93. The cut-off point with largest Youden index was 4.0, when sensitivity and specificity were 0.62 and 0.75 respectively.

According to follow-up investigation, 10 (3.6%) out of 278 the patients had DFU in the following year after the baseline. The rest 199 patients (41.7%) were lost to follow-up (no response). The followed and lost patients were not different with respect to age (*P* = 0.994), gender (*P* = 0.623), and fasting blood glucose (*P* = 0.975) at baseline. The average raw score of patients who had DFU and who had not DFU were 4.2 ± 2.3 and 2.4 ± 1.9, respectively (*P < *0.05). As shown in Fig. 3, the AUC of the ROC was 0.77 (0.61–0.93), and the cut-off point with largest Youden index was 4.0, when sensitivity and specificity were 0.62 and 0.75 respectively.

## Discussion

Our study developed and validated a DFU risk checklist among patients with diabetes from nine provinces / municipalities of China. The tool showed good construct validity and item property according to CFA and IRT results, and had measurement invariance with respect to gender, according to DIF. The risk level estimated by Bayesian method was highly correlated with Gavin’s score, indicating good concurrent validity. Internal consistency was suboptimal according to Cronbach’s alpha, which could possibly be attributed to limited number of items. Validated by one-year follow-up, the tool showed good predictive capability.

Eight items were removed from the original item pool according to the item selection criteria. Smoking was found to be inversely associated with the overall raw score and had a negative factor loading. Smoking was considered as a risk factor for ulceration because tissue hypoxia may cause vascular and neuropathic disorders in the lower extremities of diabetic patients. However, a recent meta-analysis showed that the prevalence of smoking history in patients with diabetic foot (29.1%, 95%CI: 18.3–39.6) was not significantly higher than the prevalence among those without diabetic foot (17.4%, 95%CI: 12.4–22.4)^14^. In addition, if the association of smoking with diabetic foot is mediated by vascular and neuropathic disorders, a direct evaluation of vasculopathy and neuropathy will provide more valid information of the risk. Uncomfortable shoes and socks had significant factor loadings on two or more dimensions. Inclusion of the two items will compromise the unidimensional hypothesis of IRT. In addition, the two items were relatively subjective, and did not necessarily reflect long-term wearing habits. Other items including hyperpigmentation, dry skin, rhagades, ingrown toenails and onchogryposis were also removed owing to suboptimal psychometric properties.

CFA showed an acceptable overall model fit, yet suboptimal factor loadings among several items. Since risk factors of DFU are complex, it is difficult to categorize them. We noticed that some of the dimensions such as complications was suboptimal, as it contained complications of several systems except neuropathy that was listed as a single dimension. Limited items within one dimension might impede robust estimation of parameters, especially when sample size was not very large. The IWGDF’s guideline does not provide an explicit classification of risk factor as well. The construct of the tool needs to be improved in further studies.

Modern test theory specifies the nonlinear relationships between a latent trait and item responses. In contrast to widely used logistic regression models for the prediction of an outcome, IRT allows a sample-independent estimation of model parameters. Also, logistic regression is very sensitive to multicollinearity, while IRT is free of such an issue. In addition, IRT is different with classical test theory which uses the linear accumulation of item scores as the estimation of true score. IRT uses Bayesian estimation of the latent trait based on patients’ responses to items, and it also takes measurement error into consideration. IRT has been widely used in psychology^15^ and health education^16^, while in the field of diabetes management, few studies could be found. In IRT, a discrimination parameter (*a*_*i*_) estimates how well an item could differentiate subjects with varied levels of ability. Among all items, loss of protective sensation had the highest *a*_*i*_ parameter of 1.69, which reflected a quasi-traits of clinical constructs, namely, a unipolar construct in which one end of represents the level of DFU risk, while the other pole represents its absence^15^. Meanwhile, nephropathy had the highest difficulty parameter, which indicated that patients who had a positive response to this item may be at very high risk of developing DFU.

We observed that this tool has acceptable area under the ROC curve (0.77). Generally, a tool with AUC > 70% was considered to be clinically useful^17^. When cut-off was 4, the sensitivity and specificity were 0.62 and 0.75 respectively. This cut-off was selected statistically by the Youden’s index. However, for the purpose of protecting more patients, and minimize the number of newly-occurred and recurrent DFU, we would suggest that lower threshold should be used, especially for those with a history of ulceration, although this would sacrifice specificity. Also, it might be of great interest for researchers to estimate and compare the medical cost for preventive intervention, and the cost for treatment when DFU occurs, when using different thresholds for risk management. Nevertheless, owing to the relatively low incidence of DFU (that might be attributable to our health education) observed in our study, large-scale longitudinal studies are needed to validate the tool.

The study has several limitations. First, many patients (41.7%) were lost to follow-up, although the lost patients were not different from the followed ones with respect to age, gender and fasting blood glucose at baseline. We considered this loss to follow-up as missing at random, and no major selection bias existed. Second, the study was hospital-based, and selection bias was inevitable. Third, the test-retest reliability was not determined. Last, limited number of items within a dimension resulted in suboptimal internal consistency. In spite of the limitations, the study also has strengths. First, the tool was validated using classical test theory in combination with modern test theory. The item properties were meticulously examined. Second, the study is a multi-center research, and the patients were recruited from different regions of China. The sample had sufficient variation. Third, under the IRT estimation of true scores, computer adaptive testing can be developed within mobile applications, which could further facilitate diabetes self-management. Good health management could improve the quality of life as well as clinical indicators among diabetic patients^18^. Fourth, all items in the checklist were evaluated by our nurses, and measurement error in patient-reported outcomes were minimized. Last, the predictive capability of the tool was validated by one-year follow-up.

We validated a brief diabetic foot ulceration risk checklist that is potentially useful in diabetes management in clinical (especially in multidisciplinary scenario) as well as primary health care settings. The tool had good psychometric properties as validated by classical and modern test theory. The tool also had good predictive capability as validated by one-year follow-up.
